# Jumbo phages are active against extensively drug-resistant eyedrop-associated *Pseudomonas aeruginosa* infections

**DOI:** 10.1128/aac.00654-23

**Published:** 2023-11-06

**Authors:** Ana Georgina Cobián Güemes, Pooja Ghatbale, Alisha N. Blanc, Chase J. Morgan, Andrew Garcia, Jesse Leonard, Lina Huang, Grace Kovalick, Marissa Proost, Megan Chiu, Peiting Kuo, Joseph Oh, Smruthi Karthikeyan, Rob Knight, Joe Pogliano, Robert T. Schooley, David T. Pride

**Affiliations:** 1 Department of Pathology, University of California San Diego, La Jolla, California, USA; 2 Department of Biology, University of California San Diego, La Jolla, California, USA; 3 Department of Environmental Science and Engineering, Division of Engineering and Applied Science, California Institute of Technology, Pasadena, California, USA; 4 Department of Pediatrics, University of California San Diego, La Jolla, California, USA; 5 Center for Microbiome Innovation, University of California San Diego, La Jolla, California, USA; 6 Department of Bioengineering, University of California San Diego, La Jolla, California, USA; 7 Department of Computer Sciences & Engineering, University of California San Diego, La Jolla, California, USA; 8 Howard Hughes Medical Institute, University of California San Diego, La Jolla, California, USA; 9 Department of Medicine, University of California San Diego, La Jolla, California, USA; University of Pittsburgh, Pittsburgh, Pennsylvania, USA

**Keywords:** jumbo phage, antibiotic resistance, *Pseudomonas aeruginosa*, eye drops, extensively drug resistant

## Abstract

Antibiotic-resistant bacteria present an emerging challenge to human health. Their prevalence has been increasing across the globe due in part to the liberal use of antibiotics that has pressured them to develop resistance. Those bacteria that acquire mobile genetic elements are especially concerning because those plasmids may be shared readily with other microbes that can then also become antibiotic resistant. Serious infections have recently been related to the contamination of preservative-free eyedrops with extensively drug-resistant (XDR) isolates of *Pseudomonas aeruginosa*, already resulting in three deaths. These drug-resistant isolates cannot be managed with most conventional antibiotics. We sought to identify alternatives to conventional antibiotics for the lysis of these XDR isolates and identified multiple bacteriophages (viruses that attack bacteria) that killed them efficiently. We found both jumbo phages (>200 kb in genome size) and non-jumbo phages that were active against these isolates, the former killing more efficiently. Jumbo phages effectively killed the three separate XDR *P. aeruginosa* isolates both on solid and liquid medium. Given the ongoing nature of the XDR *P. aeruginosa* eyedrop outbreak, the identification of phages active against them provides physicians with several novel potential alternatives for treatment.

## INTRODUCTION

Antibiotic-resistant bacteria present a serious and growing challenge as their numbers continue to increase across the globe (1). Most notable among those organisms whose propensity for antibiotic resistance has posed a challenge are the ESKAPE (*Enterococcus faecium*, *Staphylococcus aureus*, *Klebsiella* sp., *Acinetobacter* sp., *Pseudomonas aeruginosa*, and *Enterobacter* sp.) pathogens, which are responsible for the majority of nosocomial infections worldwide (2). These particular microbes have the potential to be MDR (multi-drug resistant; resistant to at least one antimicrobial in three different classes of antimicrobials) or XDR (extensively drug resistant; resistant to almost all approved antimicrobials), and sometimes require the use of lesser-used antibiotics for treatment or antibiotic alternatives when they cause infections in humans (3). A recent outbreak of XDR *P. aeruginosa* contaminating eye drops in the U.S. is a perfect example of the threat that such microbes may pose to the population in the long term (4, 5). Thus far, there have been at least three fatalities secondary to these *P. aeruginosa* isolates, which are incredibly difficult to treat with conventional antibiotics (6).

Many bacteria are capable of acquiring new resistance genes through the process of conjugation where they may acquire mobile genetic elements such as plasmids (7). In the current outbreak, *P. aeruginosa* has acquired the Verona integron-encoded metallo-beta-lactamase (VIM beta-lactamase), which confers resistance to carbapenem antibiotics among others (8). This renders the organism very difficult to treat with antibiotics, and because of the concern that it may be mobile, also raises significant concern for the spread of the carbapenemase to other bacteria.

Bacteriophages (viruses that attack bacteria) represent a rising therapeutic option for the treatment of antibiotic-resistant bacteria (9). Mechanisms that account for the reduced susceptibility of bacteria to antibiotics generally do not affect their susceptibilities to phages (10). Interest in the clinical use of phages for the treatment of drug-resistant bacterial infections has been kindled by favorable outcomes reported in an increasing number of case reports and case series (11, 12). In view of the ongoing eyedrop-associated *P. aeruginosa* outbreak in the U.S., we examined a collection of anti-*Pseudomonas* phages against three XDR *P. aeruginosa* isolates that were obtained from patients affected by this outbreak to identify phages capable of killing them and to identify whether they may be capable of inhibiting the XDR isolates across different conditions.

## RESULTS

### *Pseudomonas aeruginosa* isolates

We obtained each of the three separate *P. aeruginosa* isolates responsible for the ongoing outbreak found in eyedrops (3) from the CDC/FDA AR Isolate Bank (https://wwwn.cdc.gov/ARIsolateBank/; PS747, PS748, PS749, corresponding IDs in the AR bank are: 1268, 1269, and 1270). Antimicrobial susceptibility testing by microbroth dilution in the UC San Diego Center for Advanced Laboratory Medicine Clinical Microbiology Laboratory confirmed their XDR status (Table 1). The isolates were highly resistant to most beta-lactam antibiotics, including ceftolozane/tazobactam and ceftazidime/avibactam. Since the organisms possess a carbapenemase, treatment with meropenem and piperacillin/tazobactam would not be recommended despite intermediate MIC values in microbroth dilution assays. The only antibiotic to which the microbes demonstrated reproducible susceptibility was colistin (13).

**TABLE 1:** Antibiotic susceptibility of *Pseudomonas aeruginosa* epidemic isolates^*a*^

Antibiotic	PS747	PS748	PS749
Aztreonam	>16 (R)	>16 (R)	>16 (R)
Ceftazidime	>16 (R)	>16 (R)	>16 (R)
Cefepime	>16 (R)	>16 (R)	>16 (R)
Meropenem	>8 (R)	8 (I)	8 (I)
Piperacillin/Tazobactam	64 (R)	>64 (R)	32(I)
Ceftolozane/Tazobactam	>8/4 (R)	>8/4 (R)	>8/4 (R)
Ceftazidime/Avibactam	>16/4 (R)	>16/4 (R)	>16/4 (R)
Ciprofloxacin	>2 (R)	>2 (R)	>2 (R)
Levofloxacin	>4 (R)	>4 (R)	>4 (R)
Gentamicin	>8 (R)	>8 (R)	>8 (R)
Tobramycin	>8 (R)	>8 (R)	>8 (R)
Amikacin	>32 (R)	>32 (R)	>32 (R)
Colistin	≤2 (S)	≤2 (S)	≤2 (S)

^*a*^ MIC values are shown in µg/mL. Resistant (R), intermediate (I) or susceptible (S) MIC values are shown in parenthesis.

**Fig 1:**
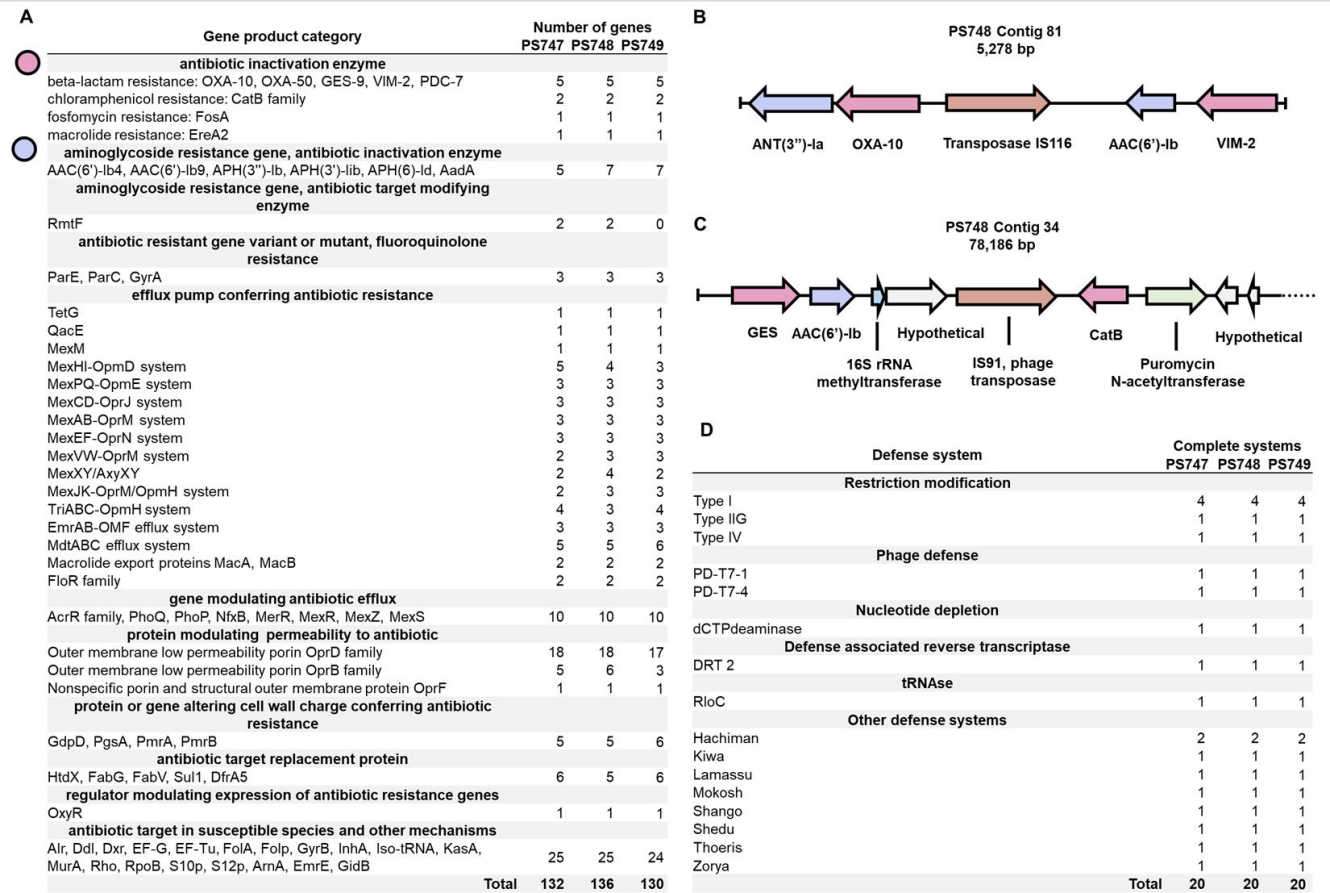
Antibiotic-resistance genes and phage defense mechanisms in eyedrop-associated *Pseudomonas aeruginosa* genomes. (**A**) Antibiotic-resistance genes. Genome annotations were performed in the BV-BCR server using the CARD database. (**B**) Selected insertion sequence (IS) element of PS748. (**C**) Selected plasmid fragment in PS748. (**D**) Identified complete phage defense systems. Identification was performed using DefenseFinder.

We also sequenced each of the *P. aeruginosa* isolates obtained from the CDC in this study. Our results were largely consistent with the sequencing of the same reported by other groups (3). We assembled the genome sequences through a combination of Illumina short-read and Nanopore long-read sequencing. We confirmed that each of the genomes was generally roughly 7 Mbp in size, with nearly 66% G + C content (Table S1). Although we were able to sequence the genomes to near completion, we were not able to assemble the genomes below 25 separate 100 Kbp contigs. Many putative antibiotic-resistance genes were identified in the genome sequences, including those associated with resistance to beta-lactam, chloramphenicol, fosfomycin, macrolides, aminoglycosides, and fluoroquinolone antibiotics (Fig. 1, panel A). The VIM beta-lactamase sequence was identified in each of the organisms. Notably, a contig of 5 Kbp carries two beta-lactamases (VIM-2 and OXA-10), two aminoglycoside resistance genes, and a transposase (Fig. 1, panel B). A plasmid of 78 Kbp carries a transposase, two beta-lactamases (GES and CatB), and several other antibiotic-resistance genes (Fig. 1, panel C). Several complete anti-phage defense mechanisms were identified in the genomes (Fig. 1, panel D) including three types of restriction-modification systems, a dCTP deaminase involved in nucleotides depletion, a defense-associated reverse transcriptase, two recently discovered phage defense systems identified in phage T7, and eight other different defense systems.

### Bacteriophages are active against XDR isolates

**Fig 2:**
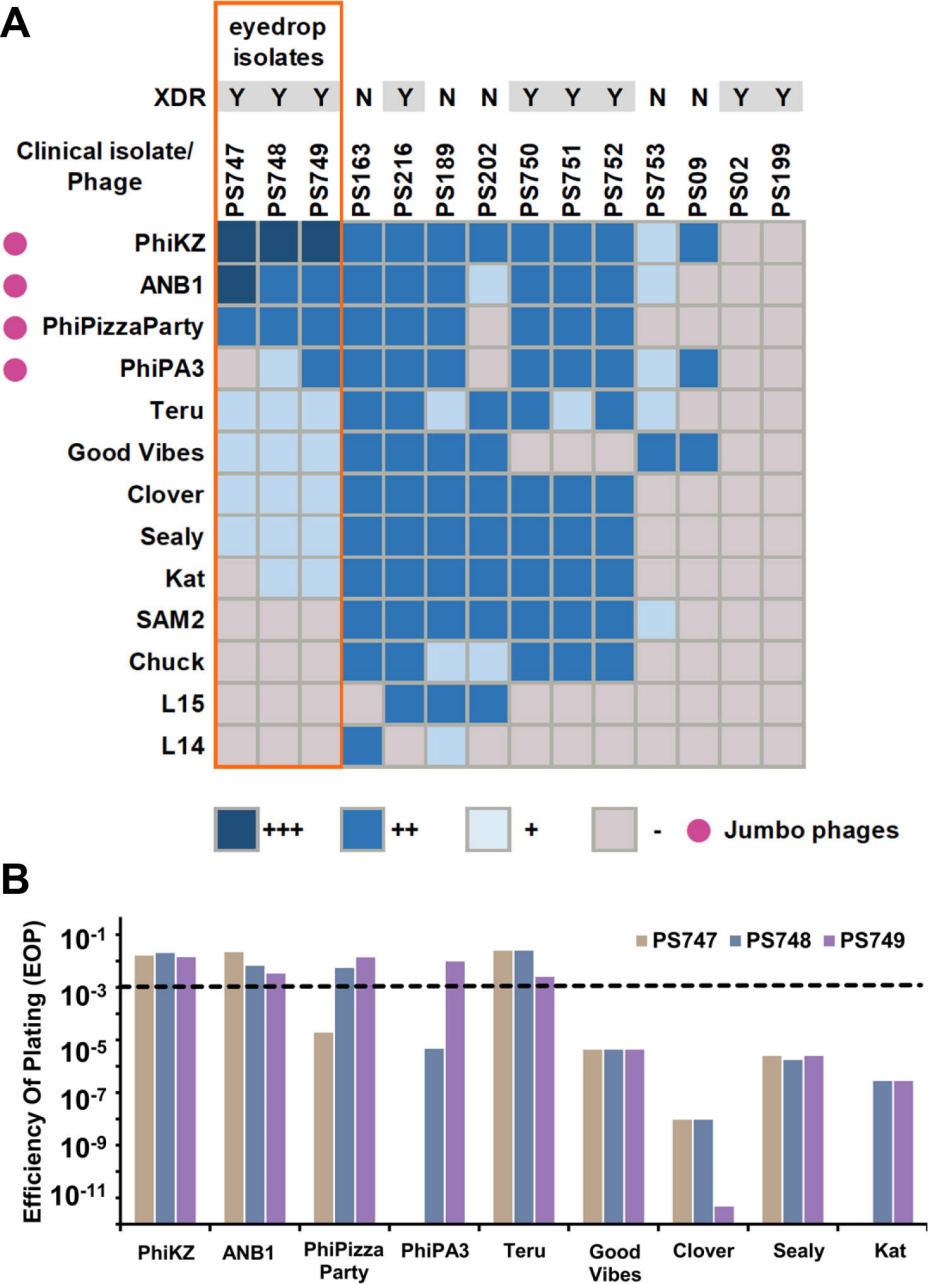
(**A**) Host range of phages in selected *P. aeruginosa* clinical isolates. +++ lysis at 1 × 10^−6^ PFU/mL, ++ lysis at 1 × 10^−5^ to 1 × 10^−3^ PFU/mL , + lysis at 1 × 10^−2^ PFU/mL to undiluted, and − no lysis. (**B**) Efficiency of plating for selected phages, *Pseudomonas aeruginosa* PAO1 was used as indicator strain. Phage titers for EOP calculations were performed in triplicate. Average EOP values are shown.

We tested a collection of phages to determine whether some were active against the XDR *P. aeruginosa* isolates and other recently identified clinical *P. aeruginosa* isolates (Table S2). We found 13 separate phages with activity against one or more of the *P. aeruginosa* isolates (Fig. 2, panel A). We first characterized the phage-host interactions using standard plaque assays on solid media. Two of the *P. aeruginosa* isolates were not lysed by any of the 13 phages in the collection. One or more phages exhibited lytic activity against each of the other 12 isolates. Nine of the thirteen phages exhibited lytic activity against one or more of the eyedrop-associated isolates, but only three phages produced clear plaques at the highest dilution tested (Fig. 2, panel A). The jumbo phages (tailed phage genomes > 200 kb) had significant lytic activity against the eyedrop isolates. These jumbo phages included the previously described PhiKZ (14) and PhiPA3 (15), but also two additional phages, ANB1 and PhiPizzaParty, which were found in this study.

### Genome characterizations and imaging of phages

**Fig 3:**
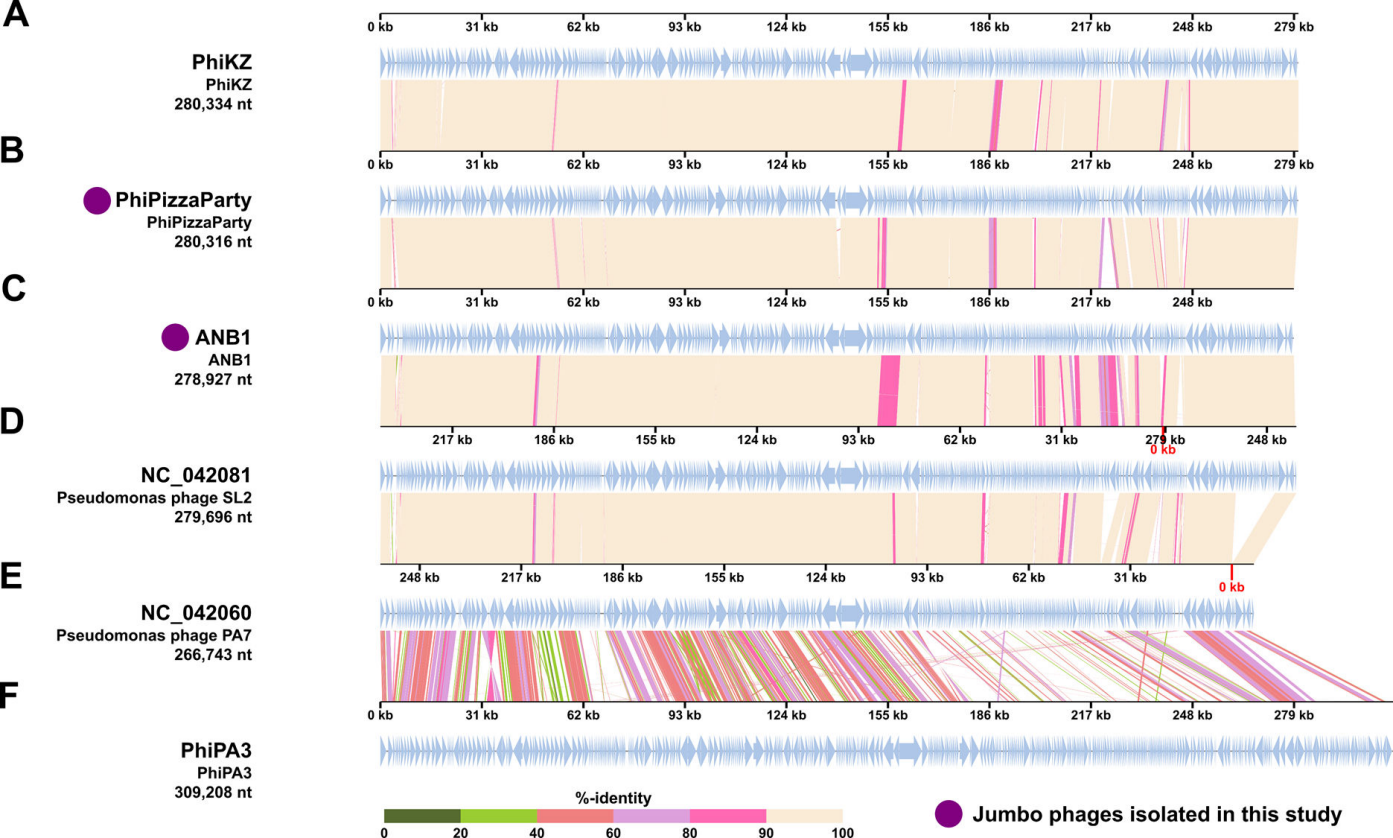
Comparative genomics of jumbo phages. Jumbo phages tested against XDR *Pseudomonas aeruginosa* eyedrop isolates (**A, B, C and F**) and its closest relatives (**D and E**). Jumbo phages PhiPizzaParty (**B**) and ANB1 (**C**) were discovered in this study. Comparative genomics were performed in the VIP tree server.

We characterized the genome and structures of the jumbo phages in this collection, which exhibited lytic activity against the outbreak-associated XDR *P. aeruginosa* isolates. Phages PhiPizzaParty and ANB1 were similar in size and gene content to PhiKZ (Fig. 3). They had many similarities and some differences along their genome structures, indicating that they were likely derived from similar ancestral phages (Fig. 3). We did not identify any gene content to suggest that they may be involved in lysogenic infections. The closest relative to PhiPizzaParty and ANB1 was phage SL2 (16), these phages have an identity greater than 97% between each other. They are substantially different from phage PA7 (NC_042060.1) and PhiPA3 (Fig. S1). Phage PhiPA3 performs generalized transduction (15); therefore, it was excluded from further analysis. Phages that perform generalized transduction are believed to be unsuitable for therapeutic applications since they could spread genes between bacteria. We found that phage PhiPizzaParty was 98.4% similar to PhiKZ, with most of the differences arising in the gene structures of endonucleases, structural head protein, tail fibers, and other proteins without annotation. Phage ANB1 was 98% similar to PhiKZ, with differences arising in similar gene structures. ANB1 was more divergent from PhiKZ than PhiPizzaParty (Fig. S1). The genomes for non-jumbo phages were also sequenced (Fig. S2).

**Fig 4:**
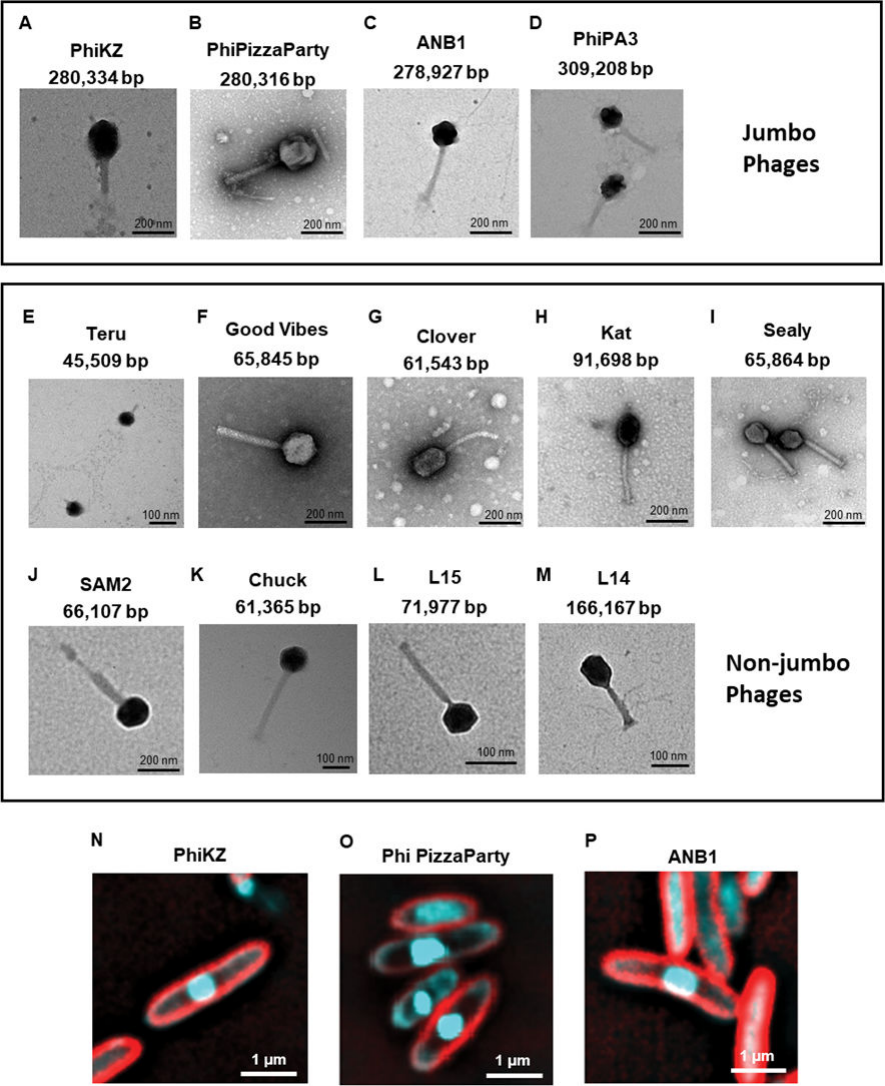
TEM of phages used in this study. (A–D) Jumbo phages. (E–M) Non-jumbo phages. (**N*–*P**) Fluorescence microscopic images of *Pseudomonas aeruginosa* infected with phage PhiKZ, Phi Pizza Party, and ANB1, respectively. The cell membrane is stained with FM4-64 (red), and the DNA is stained with DAPI (cyan). The phage nucleus-like structure can be seen centered in the cell.

We previously demonstrated that many *Pseudomonas* jumbo phages including PhiKZ replicate by enclosing their genome within a proteinaceous shell, forming a structure (phage nucleus) that segregates phage DNA from the host cell cytoplasm (17–19). Nucleus-forming phages are particularly well-suited for phage therapy because they are broadly immune to many bacterial phage defense systems (20, 21). The gene that encodes the nuclear shell protein, Chimallin (22, 23), in PhiKZ and other nucleus-forming phages was present in both PhiPizzaParty and ANB1. To determine whether these phages also formed a phage nucleus, we performed fluorescence microscopy on phage-infected cells (Fig. 4, panels N through P). Imaging of DAPI-stained infected cells showed that, similar to PhiKZ, PhiPizzaParty and ANB1 degrade the host chromosome and center its genome in the cell in a manner that is consistent with nucleus-forming phage. Together, this supports the conclusion that PhiPizzaParty and ANB1 are nucleus-forming phages.

### TEM analysis

We imaged each of the phages in this study using transmission electron microscopy (TEM) to further determine their structures. The images of each of the phages demonstrate that most have an icosahedral head structure with relatively long, noncontractile tail structures as are often observed in the family *Siphoviridae* (Fig. 4, panels A through M) (24). Phage Teru, which was also active against many of the *P. aeruginosa* isolates, was the most notable exception with more of a tail stub similar to that observed in the family *Podoviridae* (Fig. 4, panel E).

### Efficiency of plating analysis

To further characterize the activity of the phages against the XDR *P. aeruginosa* isolates, we performed an efficiency of plating (EOP) analysis in all phages that infect the eyedrop-associated isolates (Fig. 2, panel B; Table S4). Most of the non-jumbo phages produced low EOP values, with phages Good Vibes, Clover, Sealy, and Kat producing EOP values lower than 10^−3^ (Fig. 2, panel B; Table S4). Phage Teru was the only non-jumbo phage to produce EOP values similar to those of the jumbo phages; this was because Teru’s titer in the reference strain PAO1 was lower than the rest of the phages. The jumbo phages produced EOP values greater than 10^−3^ in most cases, with PhiKZ and ANB1 producing values ≥10^−3^ for each of the XDR isolates. Phage PhiPizzaParty produced EOP values ≥10^−3^ for isolates PS748 and PS749, but not for PS747.

### Liquid media suppression

**Fig 5:**
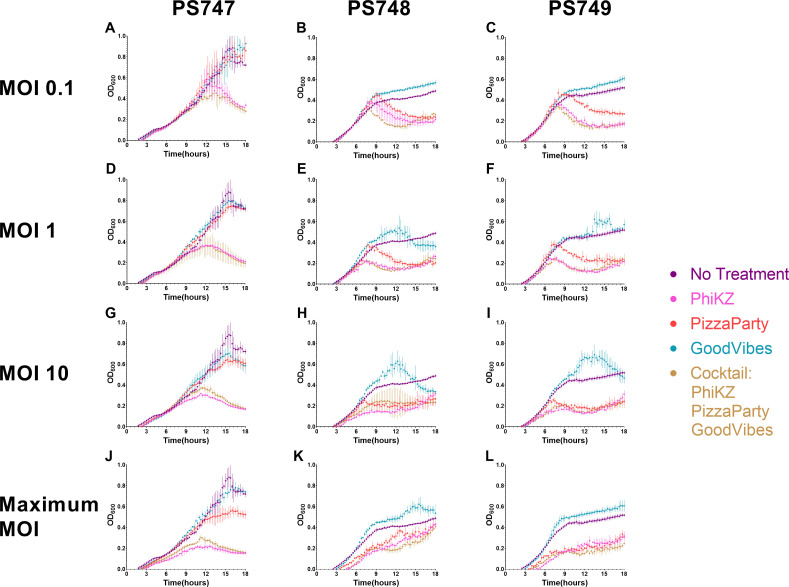
Inhibition of bacteria growth by phages in liquid media. Experiments were performed at an MOI of 0.1 (**A–C**), 1 (**D–F**), 10 (**G–I**), and maximum phage titer achievable (**J–L**). Three bacterial strains were used: *P. aeruginosa* PS747 (**A, D, G, H**), *P. aeruginosa* PS748 (**B, E, H, K**), and *P. aeruginosa* PS749 (**C, F, I, L**).

**Fig 6:**
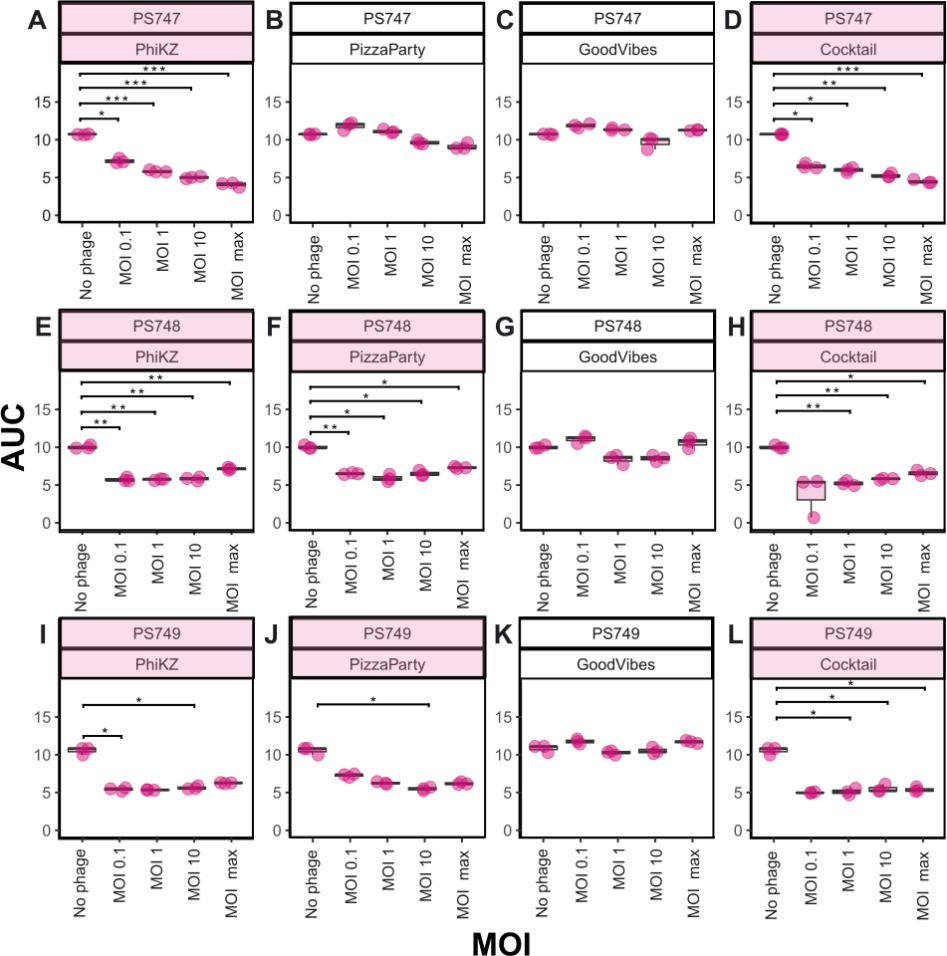
Area under the curve (AUC) measurements over different multiplicity of infection (MOI). *t*-test with multiple comparison corrections. *P < 0.05, **P < 0.01, and ***P < 0.001.

We sought to determine whether our jumbo phages also might be active in a liquid medium in addition to the solid medium against the XDR *P. aeruginosa* isolates. There was substantial inhibition of each of the XDR isolates at up to 18 hours of co-cultivation (Fig. 5). The jumbo phage PhiKZ significantly inhibits the growth of the three XDR *P. aeruginosa* isolates at all multiplicity of infection (MOI), including the lowest MOI tested of 0.1 (Fig. 6, panels A, E, and I). Jumbo phage PhiPizzaParty significantly inhibits PS748 and PS749, but does not show significant growth inhibition of PS747. The non-jumbo phage Good Vibes does not show a significant inhibition in any of the isolates regardless of the MOI used (Fig. 6, panels C, G, and K). The three XDR *P. aeruginosa* strains were significantly inhibited by a phage cocktail containing equivalent amounts of PhiKZ, PhiPizzaParty, and GoodVibes (Fig. 6, panels D, H, and L).

## DISCUSSION

We identified three jumbo phages with significant activity against three XDR *P. aeruginosa* isolates associated with a recently described multi-state outbreak among users of ophthalmic eye drops on solid and in liquid medium. We also identified a non-jumbo phage with significant activity on a solid medium. While we do not have specific information on some of these phages to describe their receptors and interactions with their host *P. aeruginosa* strains, the jumbo phages are similar to previously used phages such as OMKO1 (25), which already have been used rather extensively in phage therapy applications. The use of jumbo phages that have nucleus-like structures may have certain advantages compared to more conventional bacteriophage therapies, as the nuclear shell excludes many DNA-targeting anti-phage systems (20, 21, 26). The phages we identified in this study (ANB1, PhiPizzaParty, and Teru) were all identified from wastewater on the UCSD campus, strongly suggesting that these phages are already circulating through the population.

It is important to note that carbapenems are often the last line of therapy for the treatment of many pathogens but this is not necessarily the case for *P. aeruginosa*. This is because the organism can have multiple different mechanisms by which it can resist carbapenems (27), which may persist after the organism is cured of the VIM beta-lactamase. In many cases of *P. aeruginosa*, aminoglycoside antibiotics such as amikacin represent the last line of therapy, but in this case, each of the isolates was already resistant to aminoglycosides. These isolates present medical conundrums for therapy, as they are resistant to most commonly used antibiotics, and by the time most laboratories would be able to test for susceptibility to secondary antibiotics such as colistin and cefiderocol, the infection could become much more difficult to eradicate. The knowledge that PhiKZ (a widely available phage) has significant activity against these isolates could make it a candidate for use in phage therapy applications against these XDR *P. aeruginosa* isolates.

One of the primary concerns with the use of phages to treat bacterial infections is the potential that phages may integrate into the host genomes and cause long-term phage infections rather than immediate lysis. We did not identify any genes in either PhiPizzaParty, ANB1, or Teru, which would suggest they have the potential for lysogenic infections. PhiKZ-like phages also degrade the host chromosome early in infection further decreasing the likelihood of lysogenic infection (28, 29). Given the immense concern for the continued spread of these XDR isolates with the use of contaminated eye drops, and the relatively few antibiotics that may be effective, the development/purification of phages such as those identified here could offer critical treatment alternatives to eliminate the ongoing risk from these XDR *P. aeruginosa* strains.

## MATERIALS AND METHODS

### Bacterial strains, bacteriophages, and culture conditions

*P. aeruginosa* strains used in the study (Table S2) were previously isolated from patients with *Pseudomonas* infection at the UCSD Centre for Advanced Laboratory Medicine. The antibiotic sensitivity testing was performed on the isolates using MicroScan NM46 and DNM2 panels. The phages used in this study were previously isolated from various environmental sources (Table S3). Luria-Bertani (LB) media was used to grow the *P. aeruginosa* strains and phages at 37°C.

### Jumbo phage isolation

Wastewater was collected from the UCSD campus through the wastewater monitoring program (30, 31), and from several locations in southern California (Table S3). Jumbo phage isolation and purification were carried out as previously described (32). Wastewater was centrifuged at 3,000 × *g* for 10 min. The supernatant was collected and centrifuged at 4,696 × *g* for 55 min. The supernatant was removed, and the pellet was resuspended in 10 mL of SM buffer; this process was performed twice. The sample was treated with an equal volume of chloroform to remove residual bacteria. Plaques were purified three times in 0.3% LB agar plates. Phages were produced by plate lysis in 0.1% LB agar.

### Whole genome sequencing

Total genomic DNA from the bacteriophages and bacteria was extracted using the QIAamp UltraSens Virus kit (Qiagen catalog number 53706) or the DNeasy Blood & Tissue Kit (Qiagen catalog number 69504) respectively. Genome sequencing of phages and bacterial isolates was carried out using a paired-end approach (2 × 150 bp) on iSeq100 or Miseq platforms (Illumina), respectively. Phage sequences were assembled using *de novo* assembly algorithm of CLC Genomics Workbench (CLC Genomics, Qiagen, version 9.5.3). Rapid Annotation using Subsystem Technology pipeline (v2.0) was used to annotate the phage sequences (33). For Nanopore sequencing, bacterial DNA was extracted using the ZYMO DNA Microprep kit. Libraries were constructed using the rapid sequencing kit and sequenced on the MinION platform. Illumina and Nanopore reads were used for a hybrid assembly using SPAdes (34). Bacterial genomes were annotated using the BV-BCR (bacterial and viral bioinformatics resource center) resources (33). Antibiotic-resistance genes were obtained from BV-BCR annotations against the CARD database (35). Defense mechanisms were characterized using DefenseFinder (36).

### Host range evaluation and efficiency of plating

*P. aeruginosa* isolates listed in Table S2 were incubated overnight at 37°C. The next day, 100 µL of bacterial culture with an OD_600_ of 0.2 was mixed with 3 mL of melted LB soft agar (when soft agar temperature reaches ~45°C). This mixture was then overlaid on an LB Agar plate (1.5% Agar). A 5 µL sample of serially diluted phages and a 5  µL LB control were spotted onto the bacterial overlay, left for 30 min to dry, and then the plates were incubated overnight at 37°C. The next day, the number of plaques was counted. The EOP assays were carried out as previously described (34) using the reference strain *Pseudomonas aeruginosa* PA01 as the indicator strain. Phage titers for EOP calculations were obtained in triplicate and the average of phage titer was used for EOP calculations.

### Phage characterization using transmission electron microscopy

A carbon-coated grid (PELCO SynapTek Grids, product# 01754-F) was placed on a drop of 10 µL of freshly made phage stock (~10^10^ PFU/mL), and the grids were negatively stained with 2% uranyl acetate (pH 4.0) for 45 s. Imaging was performed using Joel 1400 plus at the University of California, San Diego—Cellular and Molecular Medicine Electron Microscopy Core (RRID: SCR_022039).

### Fluorescence microscopy of phage-infected *Pseudomonas aeruginosa*

PA01 was grown in LB to an OD_600_ of 0.5 and incubated with high titer phage lysate at a ratio of 1:100 lysate-to-culture at 30℃ with agitation for 30 min. FM4-64 dye was added to the cells at a final concentration of 6.5 µg/mL and then the culture was spotted onto a 1% agarose 25% LB pads containing 0.6 µg/mL DAPI and visualized on an Applied Precision DV Elite optical sectioning microscope with a Photometrics CoolSNAP-HQ2 camera (Applied Precision/GE Healthcare). Microscopic images were deconvolved using SoftWoRx v5.5.1. Image analysis and processing were performed in Fiji.

### Liquid phage/bacteria assays

The phage and bacterial co-cultivation assays were performed at an MOI of 0.1, 1, 10, and maximum. The bacterial cells from the exponential phase were diluted to an OD_600_ of 0.1 in fresh LB broth. Each well within 96-well plate was inoculated with 30 µL of phage + 60 µL of bacteria (OD_600_ 0.1) and the remaining volume of MacConkey media was added to a total volume of 200 µL. For the phage cocktail, 10 µL of each phage dilution was added. The OD_600_ was measured every 15 min at 37°C for 18 hours. The OD_600_ measurements were used to calculate the area under the curve (AUC) using the R package GrowthCurver (37) using the area under the logistic curve function (SummarizeGrowth, auc_l). AUC values were compared using a *t*-test, *P*-values were corrected for multiple comparisons, and calculations were made using the R package ggstatsplots (38).

## Data Availability

All sequences included in this study have been deposited in the NCBI Sequence Read Archive under BioProject PRJNA971669.

## SUPPLEMENTAL MATERIAL

Supplemental Material aac.00654-23-s0001.docx Table S1 to S5 and Fig. S1 and S2.

ASM does not own the copyrights to Supplemental Material that may be linked to, or accessed through, an article. The authors have granted ASM a non-exclusive, world-wide license to publish the Supplemental Material files. Please contact the corresponding author directly for reuse.
