# Supplementary material for: Jumbo phages are active against extensively drug-resistant eyedrop-associated Pseudomonas aeruginosa infections
Source: Antimicrob Agents Chemother. 2023 Nov 6;67(12):e00654-23. doi: 10.1128/aac.00654-23 (PMC10720484; doi:10.1128/aac.00654-23)
Supplement: Supplemental Material — Table S1 to S5 and Fig. S1 and S2. [file aac.00654-23-s0001.docx]

**SUPPLEMENTAL DATA**

|  | **PS747** | **PS748** | **PS749** | |
| --- | --- | --- | --- | --- |
| Genome length (bp) | 7,002,089 | 6,971,926 | 6,973,184 | |
| Contigs > 100 kb | 21 | 25 | 16 | |
| Contigs > 1 kb | 123 | 102 | 191 | |
| Total contigs | 142 | 186 | 215 | |
| CheckM completeness | 98.6 | 98.4 | 98.4 | |
| GC content | 65.9 | 65.9 | | 65.8 |
| GenBank Accession | SAMN35051892 | SAMN35051893 | | SAMN35051894 |

**Table S1.** *P. aeruginosa* genomes sequenced in this study.

**Table S2.** *P. aeruginosa* strains used in the study.

| ID | Species | Isolation source | Phenotype | Antimicrobial resistance |
| --- | --- | --- | --- | --- |
| PS747 | *P. aeruginosa* | Eyedrops | mucoid | Aztreonam, Ceftazidime, Piperacillin/Tazobactam, Ciprofloxacin, Cefepime, Amikacin, Gentamicin, Tobramycin, Meropenem, Ceftolozane/Tazobactam, Ceftazidime/Avibactam, Levofloxacin |
| PS748 | *P. aeruginosa* | Eyedrops | mucoid | Aztreonam, Ceftazidime, Piperacillin/Tazobactam, Ciprofloxacin, Cefepime, Amikacin, Gentamicin, Tobramycin, Meropenem, Ceftolozane/Tazobactam, Ceftazidime/Avibactam, Levofloxacin |
| PS749 | *P. aeruginosa* | Eyedrops | mucoid | Aztreonam, Ceftazidime, Piperacillin/Tazobactam, Ciprofloxacin, Cefepime, Amikacin, Gentamicin, Tobramycin, Meropenem, Ceftolozane/Tazobactam, Ceftazidime/Avibactam, Levofloxacin |
| PS163 | *P. aeruginosa* | throat from CF patient | mucoid | no resistance detected |
| PS216 | *P. aeruginosa* | sputum from a CF patient | non mucoid | Aztreonam, Ciprofloxacin, Cefepime, Piperacillin/Tazobactam |
| PS189 | *P. aeruginosa* | sputum from a CF patient | non mucoid | no resistance detected |
| PS202 | *P. aeruginosa* | urine | non mucoid | no resistance detected |
| PS750 | *P. aeruginosa* | sputum from a CF patient | mucoid | Aztreonam, Ceftazidime, Piperacillin/Tazobactam, Ciprofloxacin, Cefepime, Amikacin, Gentamicin, Tobramycin, Meropenem, Ceftolozane/Tazobactam, Ceftazidime/Avibactam, Levofloxacin |
| PS751 | *P. aeruginosa* | sputum from a CF patient | mucoid | Aztreonam, Ceftazidime, Cefepime, Amikacin, Gentamicin, Piperacillin/Tazobactam, Meropenem, Ceftolozane/Tazobactam, Ceftazidime/Avibactam, Levofloxacin |
| PS752 | *P. aeruginosa* | sputum from a CF patient | mucoid | Aztreonam, Ceftazidime, Piperacillin/Tazobactam, Ciprofloxacin, Cefepime, Amikacin, Gentamicin, Tobramycin, Meropenem, Ceftolozane/Tazobactam, Ceftazidime/Avibactam, Levofloxacin |
| PS753 | *P. aeruginosa* | sputum | non mucoid | Aztreonam, Ceftazidime, Cefepime |
| PS09 | *P. aeruginosa* | sputum | non mucoid | no resistance detected |
| PS02 | *P. aeruginosa* | sputum from a CF patient | mucoid | Aztreonam, Ceftazidime, Ciprofloxacin, Cefepime, Amikacin, Meropenem, Tobramycin, Piperacillin/Tazobactam, Ceftolozane/Tazobactam, Colistin |
| PS199 | *P. aeruginosa* | urine | non mucoid | Amikacin, Aztreonam, Ciprofloxacin, Cefepime, Gentamicin, Meropenem, Tobramycin, Piperacillin/Tazobactam |

**Table S3.** Isolation source and genome length of phages used in this study.

| **Phage** | **GenBank**  **accession** | **Genome size (bp)** | **Phage isolation source** | |
| --- | --- | --- | --- | --- |
| PhiKZ | NC_004629.1 | 280,320 | Sputum from COPD patient | |
| PhiPizzaParty | OQ992562 | 280,316 | UCSD campus wastewater, CA | |
| ANB1 | OQ992561 | 278,920 | | UCSD campus wastewater, CA |
| PhiPA3 | NC_028999.1 | 309,208 | | Sewage from Milton, Cambridge, UK |
| Teru | OQ992551 | 45,500 | | UCSD campus wastewater, CA |
| Good vibes | OQ992552 | 65,845 | | Tijuana, Mexico wastewater |
| Clover | OQ992553 | 61,543 | | UCSD campus wastewater, CA |
| Kat | OQ992554 | 91,698 | | Fallbrook wastewater, CA |
| Sealy | OQ992555 | 65,864 | | Point Loma wastewater, CA |
| Chuck | OQ992557 | 61,365 | | Orange county wastewater, CA |
| L14 | OQ992558 | 166,167 | | Orange county wastewater, CA |
| L15 | OQ992559 | 71,977 | | UCSD campus wastewater, CA |
| SAM2 | OQ992560 | 66,107 | | Point Loma wastewater, CA |
|  |  |  | |  |

**Table S4.** Efficiency of plating. Phage titers were performed in triplicate. The average titer values are shown.

| **Phage** | **Titer on PA01** | **Titer on PS747** | **Titer on PS748** | **Titer on PS749** | **EOP PS747** | **EOP PS748** | **EOP PS749** |
| --- | --- | --- | --- | --- | --- | --- | --- |
| PhiKZ | 1x10^10^ | 1.6x10^8^ | 2x10^8^ | 1.4x10^8^ | **1.6x10^-2^** | **2x10^-2^** | **1.4x10^-2^** |
| ANB1 | 6x10^9^ | 1.3x10^8^ | 4x10^7^ | 2x10^7^ | **2.1x10^-2^** | **6.6X10^-3^** | **3x10^-3^** |
| PhiPizzaParty | 7.3x10^9^ | 1.4x10^5^ | 4x10^7^ | 1x10^8^ | 1.9x10^-5^ | **5.4X10^-3^** | **1.3x10^-2^** |
| PhiPA3 | 1.7x10^9^ | 0 | 8x10^3^ | 1.6x10^7^ | 0 | 4.6x10^-6^ | **9.6x10^-3^** |
| Teru | 8x10^6^ | 2x10^5^ | 2x10^5^ | 2x10^4^ | **2.5X10^-2^** | **2.5X10^-2^** | **2.5X10^-3^** |
| Clover | 2.1x10^11^ | 2x10^3^ | 2x10^3^ | 1x10^0^ | 9X10^-9^ | 9X10^-9^ | 4X10^-12^ |
| Sealy | 8x10^8^ | 2x10^3^ | 1.4x10^3^ | 2x10^3^ | 2.5X10^-6^ | 1.7X10^-6^ | 2.5X10^-6^ |
| Good Vibes | 4.6x10^8^ | 2x10^3^ | 2x10^3^ | 2x10^3^ | 4.2X10^-6^ | 4.2X10^-6^ | 4.2X10^-6^ |
| Kat | 2.8x10^10^ | 0 | 8x10^3^ | 8x10^3^ | 0 | 2.7x10^-7^ | 2.7x10^-7^ |
| EOP values above 1x10^-3^ are considered effective. | | | | | | | |

**Table S5.** Closest genomes to non-jumbo phages.

| **Phage** | **NCBI Blastn results** | **GeneBank ID** | **Query cover (%)** | **Identity (%)** | **Predicted morphology** | **Observed morphology** | **Genus** |
| --- | --- | --- | --- | --- | --- | --- | --- |
| Teru | Pseudomonas phage vB_PaeP_FBPa29, complete genome | ON857936.1 | 91 | 96.12 | Podovirus | Podovirus | Bruynoghevirus |
|  | Pseudomonas phage vB_PaeP_C2-10_Ab22, complete genome | NC_026599.1 | 98 | 95.74 | Podovirus | Podovirus | Bruynoghevirus |
|  | Pseudomonas phage Epa4, complete genome | MT118288.1 | 95 | 97.84 | Podovirus | Podovirus | Bruynoghevirus |
| Good vibes | Pseudomonas phage 771-2, complete genome | OQ319932.1 | 99 | 98.14 | Myoviridae | Myoviridae | Pbunavirus |
|  | Pseudomonas phage 6917, complete genome | OL362268.1 | 99 | 97.69 | Myoviridae | Myoviridae | Pbunavirus |
|  | Pseudomonas phage 6959, complete genome | OL362269.1 | 99 | 97.6 | Myoviridae | Myoviridae | Pbunavirus |
| Clover | Pseudomonas phage PSA20, partial genome | MZ089734.1 | 95 | 97.33 | Siphoviridae | Myoviridae | Yuavirus |
|  | Pseudomonas phage vB_PaeS_S218, complete genome | MF490239.1 | 97 | 97.32 | Siphoviridae | Myoviridae | Yuavirus |
|  | Pseudomonas phage Psa21-HRN, partial genome | MZ089735.1 | 95 | 97.16 | Siphoviridae | Myoviridae | Yuavirus |
| Kat | Pseudomonas phage 908-1, complete genome | OQ319933.1 | 95 | 99.09 | Myoviridae | Myoviridae | Pakpunavirus |
|  | Pseudomonas phage vB_PaM_EPA1, complete genome | NC_073609.1 | 93 | 96.9 | Myoviridae | Myoviridae | Pakpunavirus |
|  | Pseudomonas phage 20Sep416, complete genome | NC_073612.1 | 95 | 97.22 | Myoviridae | Myoviridae | Pakpunavirus |
| Sealy | Pseudomonas phage phiLCL12, complete genome | OQ428192.1 | 96 | 96.79 | Myoviridae | Myoviridae | Pbunavirus |
|  | Pseudomonas phage 6959, complete genome | OL362269.1 | 99 | 97.23 | Myoviridae | Myoviridae | Pbunavirus |
|  | Pseudomonas phage Kara-mokiny kep-wari Wadjak 8, complete genome | OP310974.1 | 99 | 97.15 | Myoviridae | Myoviridae | Pbunavirus |
| Chuck | Pseudomonas phage M6, complete genome | NC_007809.1 | 96 | 97.9 | Siphoviridae | Siphoviridae | Yuavirus |
|  | Pseudomonas phage Psa21-HRN, partial genome | MZ089735.1 | 96 | 97.49 | Siphoviridae | Siphoviridae | Yuavirus |
|  | Pseudomonas phage PAE1, complete genome | NC_028980.1 | 94 | 98.02 | Siphoviridae | Siphoviridae | Yuavirus |
| L14 | Bacteriophage sp. isolate 3387_85790, partial genome | OP072608.1 | 97 | 97.84 | - | - | - |
|  | Escherichia phage 4E10, complete genome | OQ689735.1 | 94 | 96.12 | Myoviridae | Myoviridae | Tequatrovirus |
|  | Shigella phage JK23, complete genome | MK962752.1 | 97 | 96.97 | Myoviridae | Myoviridae | Tequatrovirus |
| L15 | Pseudomonas phage YH6, complete genome | KM974184.1 | 97 | 94.59 | Schitoviridae | Myoviridae | Litunavirus |
|  | Pseudomonas phage LP14, complete genome | MH356729.1 | 98 | 94.03 | Schitoviridae | Myoviridae | Litunavirus |
|  | Pseudomonas phage Pa2, complete genome | NC_027345.1 | 98 | 93.47 | Schitoviridae | Myoviridae | Litunavirus |
| SAM2 | Pseudomonas phage zikora, complete genome | MW557846.1 | 98 | 96.93 | Myoviridae | Myoviridae | Pbunavirus |
|  | Pseudomonas phage misfit, complete genome | MT119367.1 | 98 | 96.95 | Myoviridae | Myoviridae | Pbunavirus |
|  | Pseudomonas phage PA19, complete genome | OP831167.1 | 98 | 97.03 | Myoviridae | Myoviridae | Pbunavirus |

**Figure S1.** Identity amongst the jumbo phages presented in this work.


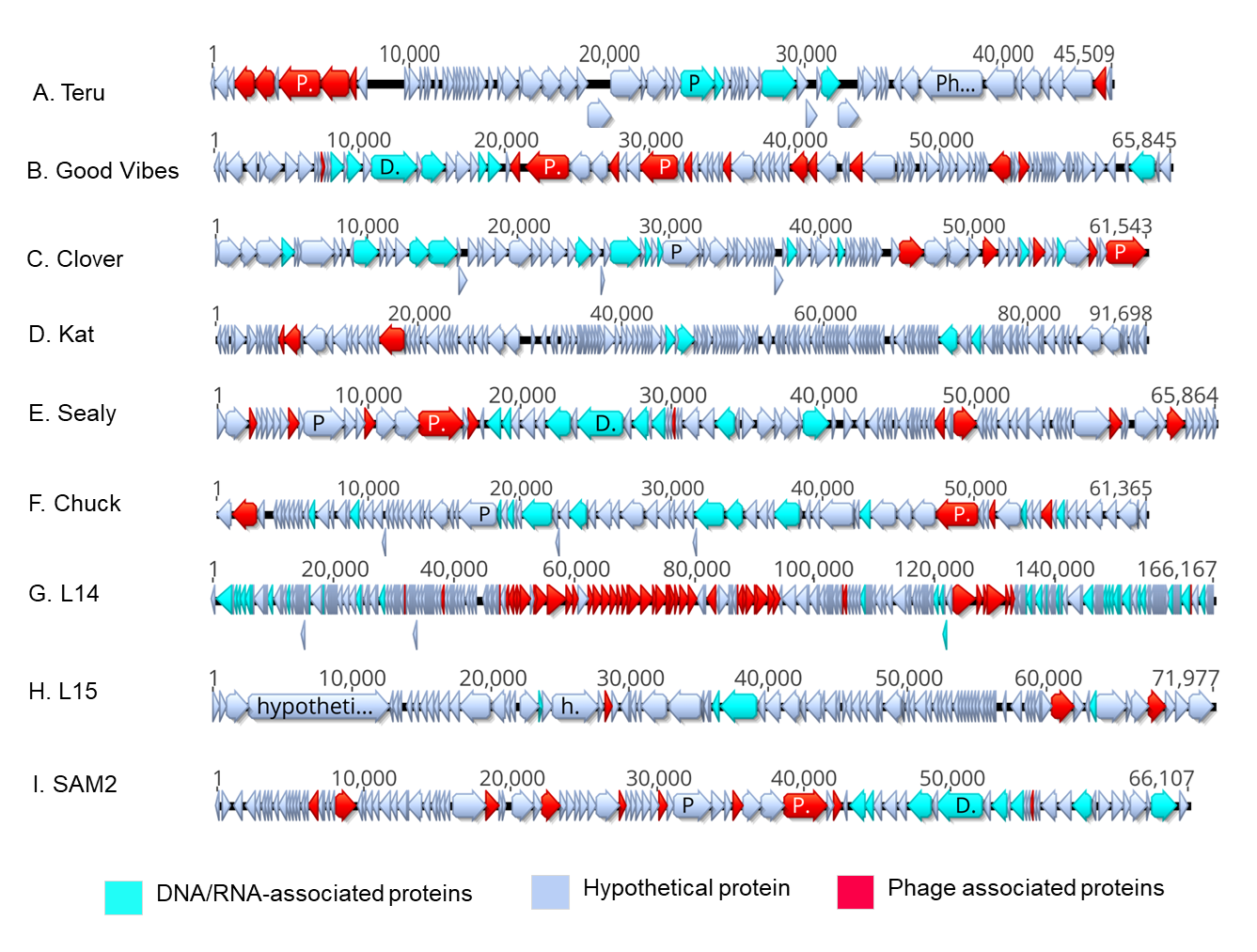


**Figure S2**. Genome annotation of non-jumbo phages characterized in the study.
